# Intersectionality at Work: South Asian Muslim Women’s Experiences of Employment and Leadership in the United Kingdom

**DOI:** 10.1007/s11199-017-0741-3

**Published:** 2017-03-25

**Authors:** Memoona Tariq, Jawad Syed

**Affiliations:** 1 The Business School, University of Huddersfield, Queensgate, Huddersfield, HD1 3DH UK; 2 Suleman Dawood School of Business, Lahore University of Management Sciences, Lahore, 54792 Pakistan

**Keywords:** Ethnicity, Gender, Intersectionality, Leadership, Muslim women, Religion, South Asian heritage

## Abstract

Drawing on qualitative interviews with 20 South Asian heritage, Muslim, female leaders, managers, and supervisors in the United Kingdom, we examine the multi-layered issues and challenges they face in pursuit of employment and leadership positions. The paper offers an intersectional perspective taking into account interconnected and overlapping factors (gender, ethnicity, religion, and family status) that affect not only the issues and challenges these women face in the labour market but also the individual agency and strategies they use to overcome any obstacles in the way of their employment and career. The results show that although Muslim women continue to face a myriad of challenges in the workplace, they are also able to tackle some of these issues through their individual strategies and networks, such as personal networks and further education. The study highlights the need for policymakers and employers to consider intersectionality to enable ethnic minority women’s inclusion and leadership within and outside the workplace.

**Electronic supplementary material:**

The online version of this article (doi:10.1007/s11199-017-0741-3) contains supplementary material, which is available to authorized users.

Despite equal opportunity legislation and other institutional interventions, ethnic minority women remain under-represented and disadvantaged in employment and leadership positions in UK organisations (Goddard 2013; Heath and Martin 2013). For example, an analysis of 10,000 Financial Times Stock Exchange (FTSE) 100 executives suggests that a glass ceiling is still in place, with only a dozen of the 289 top posts of chief executive, chairperson, or chief financial officer held by women, as well as just ten by ethnic minorities. More than half of these companies have no Leaders of Colour on the board, and two-thirds have no full-time executives from ethnic minority groups at board level. Although women occupy about one in four of the top 100 positions across FTSE 100 companies, leaders of ethnic minority origin account for just under one in 17 and only a fraction of them are ethnic minority women (Asian Image 2014; Groom 2014). This is a major concern for the UK government and employers because while women and ethnic minorities are under-represented, companies continue to face skill shortages and inequality (Grodach and Silver 2013).

However, barring a few notable exceptions (e.g., Kamenou and Fearful 2006; Littrell and Nkomo 2005; Paludi 2013; Syed and Murray 2009; Verbeek and Groenveld 2012), issues and challenges facing female ethnic minority employees remain under-explored in academic and practitioner literature (Cornelious 2002; Shen et al. 2009). In particular, there is a need to investigate how ethnic minority women are perceived and treated in organisational approaches and practices of leadership, and how the prevalent approaches affect their participation in employment and leadership. Although scholars have generally examined different styles and practices of leadership within organisations, less attention has been paid to this topic by taking intersectionality into account (Okozi et al. 2014; Sanchez-Hucles and Davis 2010). In particular, ethnic minority women seem to be relatively ignored in scholarship and practices of leadership. To address this gap, the present paper focuses on the following research question: What are the implications of intersectionality for South Asian Muslim women’s experiences of employment and leadership in organisations in the United Kingdom. Drawing on in-depth interviews with Muslim women in leadership, the present paper highlights how these women are affected by intersectionality of their gender, ethnicity, religion, and other dimensions of their identity at work.

## Intersectionality at Work

We use intersectionality theory (Crenshaw 1989) in our paper to examine employment- and leadership-related issues, experiences, and perceptions of South Asian heritage Muslim women at work. *Intersectionality* relates to how two or more dimensions of identity (such as gender, ethnicity, and religion) may result in multiple and intertwined layers of discrimination or disadvantage (Syed and Özbilgin 2015). In other words, intersectionality theory examines how gender, ethnicity, religion, and other dimensions of identity are interconnected with one another and cannot be differentiated.

Given the social construction of gender and race, and also in view of the concurrent rise of Islamic radicalism and Islamophobia in the west, it is likely that Muslim women in western workplaces are subjected to complex challenges related to their gender, ethnicity, religion, and other individual dimensions of identity, such as family status and qualification(s) (Brah 1994; Koopmans 2015). A focus on one category alone does not offer a holistic and realistic picture of the challenges which may impede Muslim women’s careers and aspirations for leadership. An intersectional lens is helpful to draw attention to the way “various socially created categories interact in intersecting systems of oppression” (Turner 2011, p. 345). It helps in analysing how gender, race, and class intersect with one another, producing different experiences for women from different race and classes (Chow et al. 2011; Shields 2008). It can also be used to look at other intersections, such as sexuality, religion, disability, age, and nationality (Turner 2011).

## Employed Muslim Women

In the United Kingdom, Muslims represent about 5% of the population, and Islam is currently the second fastest growing religion (Iqbal 2016). However, due to a host of reasons, not least of which are negative stereotypes of Islam in the aftermath of 9/11 [the bombing of the World Trade Center in the United States in 2001] and 7/7 [London bombings in July 2005], British Muslims are reported to experience job discrimination that is worse than for any other ethnic minority group. Government statistics suggest that British Muslims in general, particularly Muslim women, have the highest unemployment rates in the United Kingdom (House of Commons 2016).

A report published by the Department for Work and Pensions shows that Pakistani and Bangladeshi heritage women have the highest unemployment rates (9.8%) followed by Chinese (9.5%) and Indian (5.4%), whereas White British men (4.9%) and women (4.5%) have the lowest unemployment rates in the United Kingdom (Delebarre 2016). It may be noted that the unemployment rate refers to the percentage of the total labour force that is unemployed but actively seeking employment and willing to work. This shows that South Asian Muslim women are still struggling to gain employment and equality in the workplace. In addition to gender and ethnicity, there is an issue of intersection of gender and religion. Dobson (2014) suggests that Muslim women are up to 65% less likely to be employed than are White Christian women of same age and qualifications. This comparison shows that ethnic minorities, especially Muslim women, are still struggling to even gain employment.

Muslim women are likely to face far more challenges and discrimination based on their intersectionality than are women from majority and other ethnic backgrounds (Wallis and Robb 2013). For example, Muslim women who wear the hijab are more easily identifiable in terms of their faith and hence likely to face discrimination in the workplace (Kumra and Manfredi 2012). Discrimination has also been identified at the recruitment and selection stage. For example, Muslim women have reported being questioned about their intentions with regards to marriage and children (Wallis and Robb 2013). Although this may apply to all women, some employers assume that Muslim women are likely to leave employment soon after they are married (Ahmed and Sardar 2012).

## Participation in Management and Leadership

Despite the fact that an increasing number of South Asian Muslim women have college- or university-level qualifications, they still remain under-represented in British organisations (Paludi 2013; Platt 2009). Although, the government is encouraging organisations to take diversity into account, there is a concern that ethnic minorities, particularly women, remain under-represented at the board level (Bailey et al. 2015). According to an estimate, 62% of FTSE 100 boards are all-White. Only 5.25% of board members are from ethnic minorities compared to 14% of the British population (Guibourg 2014).

Previous studies suggest that one of the reasons why ethnic minority women, particularly those from South Asian heritage, are under-represented in organisations is because they may not have the relevant skills, education or even be fluent in the English language (Fearful and Kamenou 2010; Syed and Murray 2009). However, Bailey et al. (2015) argue that an increasing number of Muslim women are becoming well educated and are more ambitious than those women from other ethnic minority groups. Instead these women face difficulties in being hired or promoted due to strong biases against their ethnicity and religion, as well as negative gender stereotyping they receive from men including those of their own background (Farrar et al. 2012).

According to the UK census data, a key obstacle that South Asian Muslim women face while trying to progress in their careers is the social element at work (Contractor 2012). Muslims, particularly women, cannot participate in certain topics of office conversations (e.g., around non-marital relationships and alcohol) due to cultural and religious reasons (Contractor 2012). Also, social get-togethers at pubs and Christmas parties may be challenging due to the presence of alcohol. This means that Muslim women may feel isolated and miss out on important networking and social opportunities that are usually quite vital for promotions. This has caused some of these women to seek support outside their work whereas a few of them feel the burden of having to adopt the western culture (Bano 2012).

## Ethnicity and Religion

Islamic faith requires modest dressing for women, particularly in the public space (Kabir 2010). However, in certain organisations women are expected to dress in a particular way (Ahmed and Sardar 2012), such as by wearing a skirt and blouse. Occasionally, Muslim women are frowned upon by senior colleagues and employees for their choice of clothing, in particular, the hijab (Jones 2013). For example, Ms. Farrah (employee) brought a claim forward for unfair dismissal and direct religious discrimination against her former employer, Global Luggage Co, because when she started to wear the hijab, the company moved her to a different store to maintain its “trendy” image. A couple of months later, she was forced to resign after she took an extended lunch break. The employment tribunal ruled that the company had seized an incident of misconduct as a pretext for dismissing her and that the real reason for her forced resignation was that she wore the hijab to work (Furness 2012). This suggests that cultural challenges still affect Muslim women in a negative manner.

However, some employers still see religion as a nuisance and are unwilling to accommodate ethnicity or religious diversity at work (Ahmed and Sardar 2012). With regard to cultural demands, some organisations hire individuals who fit with the organisational culture rather than taking their skills, knowledge, and qualifications into account (Zempi and Chakraborti 2014). This means that the employer would choose someone on the basis that they fit with the organisation’s norms and these are usually based on the western culture, notwithstanding how well the individual is qualified and experienced for the job. When Islamic religion is such a taboo form of identity, some Muslim women may prefer to remain unemployed rather than being frowned upon or perceived as violating their own religious norms (Weller 2011). Dobson (2014) argues that a key reason why Muslims, especially Muslim women, continue to face complex challenges in the labour market in Britain is due to the growing issue of Islamophobia.

*Islamophobia* refers to the fear of Islam and hostility that Muslims experience from society (Morey and Yaqin 2011). An analysis of the labour force survey conducted by the UK’s Office for National Statistic found that Islamophobia exists in the workplace (House of Commons 2016). The analysis found that ethnicity and race made a small difference to whether individuals were hired or not. However, findings indicated a growing issue of Islamophobia and intolerance (Triandafyllidou 2010) toward Muslims, especially Muslim women; employers were less likely to hire them or even consider them for senior positions (Ridley 2014). According to the statistics published by the Metropolitan Police, anti-Muslim hate crime in Britain has risen by 70% since 2014. “Tell Mama,” an organisation that monitors Islamophobic attacks, states that 60% of such attacks are directed at Muslim women due to the reason that at least some of them are easily identifiable (e.g., due to their hijab or other traditional Islamic clothing; Sanghani 2015).

## Family Issues

Not unlike other women, Muslim women have been traditionally responsible for the caring duties at home, such as bringing up their children and looking after the extended family (Wood et al. 2012). This is still the case in some Muslim families, and often these women are expected to take the back seat in their career once they are married and have children (Ahmed and Sardar 2012; Hopkins and Gale 2009). In fact, some Muslim families fear that a working woman may bring shame on them as a public expression of the inability of the husband to provide sufficiently for his family (Hopkins and Gale 2009). Lovat (2012) argues that women who decide to take paid employment often seek jobs that offer flexibility and less workload and stress and that are mainly part-time with low prospects for leadership and growth. In addition, Muslim women with professional careers are often found in low-tier jobs once they are married and have children (Lovat 2012).

## Women’s Agency and Strategies

Recent research suggests that some ethnic minority women are not simply voiceless victims but rather are active agents towards change (Hargreaves and Anderson 2014). Some of these women use their agency to address issues that are of concern not only to themselves but also to the entire community (Hargreaves and Anderson 2014). *Agency* refers to an individual’s ability to make effective choices and to transform those choices into desired outcomes (Kabir 2010). Agency is a process through which women use their unique resources and endowments, as well as take advantage of available opportunities, to achieve their desired outcomes.

However, Afshar (2012) claims that many Muslim women still lack self- confidence and self-esteem, even those who are well-educated and articulate. Generally, Muslim women find it uncomfortable to apply for senior or leadership roles within organisations because they consider themselves lacking in skills and expertise to become successful leaders (Kabir 2010). Further concern is that many Muslim women tend to be undermined by their male counterparts because these women are perceived to be less outspoken in the boardroom (Ghumman and Ryan 2013). In contrast, those women who are successful in holding a senior or leadership position engage in number of strategies, such as networking, mentoring, coaching, and taking training development courses (Hargreaves and Anderson 2014).

There are, however, examples of ethnic minority women who have been successful in their careers (Hargreaves and Anderson 2014). Grine (2014) notes that some of these women make good use of networking and mentoring opportunities, which they tend to do in their own time. Grine also reports a case of a Muslim female teacher who was active and hardworking but faced problems in the workplace, particularly from her co-workers. Through appropriate coaching, she became self-confident and empowered, which helped her seek new opportunities while furthering in her career. Her case is an example of agency because this woman shows how she was able to progress in her career.

Indeed, it is not always the ethnic majority or White colleagues who engage in discrimination. In an interesting case reported in 2010, an employment tribunal was held because a Muslim woman was dismissed by her employer for *not* choosing to wear a headscarf to work. The case is particularly interesting because both Mr. Ghafoor (the employer), and Miss Khan (the employee who suffered discrimination) are both Muslims. The tribunal found that Miss Khan had suffered religious discrimination due to her lack of belief that Islam requires women to wear a headscarf. Miss Khan also succeeded in claiming sex discrimination because one of her colleagues stated that Mr. Ghafoor previously said he would have preferred to work with a man, but not with a woman who refused to wear a headscarf (Read 2010). The case shows that Muslim women are prepared to use their agency and strategies to overcome the complex issues and challenges that affect their career.

The foregoing literature has highlighted the multi-layered issues that South Asian heritage Muslim women face in gaining employment and leadership positions in UK organisations. However, there is a need to develop an in-depth understanding of the challenges and strategies that South Asian Muslim women use while progressing to leadership positions. Thus, we focus on the following research question: What are the implications of intersectionality for South Asian Muslim women’s experiences of employment and leadership in organisations in the United Kingdom?

## Method

### Participants

All 20 participants were Muslim women from South Asian heritage who were in a managerial or a leadership position such as chief executive officer, company director, entrepreneur, or senior manager. All participants were recruited using personal contacts, social networks such as LinkedIn and Twitter, and community events, such as the Muslim Awards 2015. Also a snowball sampling method was used to gain further access to these women. The snowball method is particularly appropriate when the members of a special population are difficult to locate or access (Ruben and Babbie 2009).

Fourteen of 20 participants were married and six were unmarried; three were 20–29 years-old; ten, 30–44 years-old; and seven, 45–59 years-old. They worked in diverse sectors such as banking, education, charity, retail, and technology. Almost all participants were in full-time positions for more than a year. Eight were categorized as leaders (holding a chief executive role or an entrepreneurial position); eight, as managers (holding a middle-to-senior management position); and four, as supervisors (being in a responsible position but at the lower end of the organisational hierarchy).

### Procedure and Materials

We adopted a qualitative approach to develop a nuanced understanding of issues and challenges South Asian Muslim women face in pursuit of employment and leadership positions in the United Kingdom. The qualitative approach is useful to “explore and understand the meaning individuals or groups ascribe to a social or human problem” (Creswell 2014, p. 4), providing a rich description based on one’s experience (Lapan et al. 2012). Participants in our study were asked questions about the extent to which their gender, ethnicity, religion, and family affected them in achieving leadership positions. The semi-structured interviews focused on the extent to which their identity affect them in achieving a managerial or leadership role in the organisation. Questions also focused on individual agency and strategies these women used to overcome such issues and challenges. In particular, the following questions were asked: “To what extent has your gender, ethnic background or/and religion affected you in achieving senior, managerial or leadership roles within your organisation?”; “How did you overcome such issues and challenges?”; and “What kind of support did you receive from your organisation to develop yourself as a manager or leader?” (The full interview guide is available as an online supplement.)

A pilot interview was conducted with two participants to refine the research instrument. Accordingly, amendments were made to the original interview schedule and further interviews were conducted. The data were collected using semi-structured interviews. Several contact methods were used, such as face-to-face, telephone, and Skype to conduct these interviews. A total of 20 interviews were conducted, each lasting from 1 to 1 1/2 h. The number was deemed enough due to iterative nature of the analysis and saturation of themes. It is, however, a fact that there are very few Muslim women from South Asian heritage that represent managerial and leadership positions. Most Muslim women are either economically inactive or working at the lower end of the organisational hierarchy. Thus, identifying and then securing the co-operation of the women we recruited was not an easy task.

Fourteen of our 20 interviews were digitally recorded via audio-transcriptions; the other six participants did not wish to be recorded. Some of these women were a bit concerned that if identified, they might face issues in their families. They were, however, assured that all information would be treated in a strictly confidential manner. Detailed written notes were taken, and transcripts of the final notes were emailed to the participants asking them to review, confirm, and amend the content as appropriate. Where needed, follow-up questions were asked to probe and clarify relevant issues. The interviews were conducted through a private, one-to-one conversation with a UK-born, 26–35 year-old, female interviewer of the same ethnic and religious heritage. In line with Glassner and Loughlin (1987), a rapport was built with each participant by displaying the interviewer’s genuine interest in understanding the interviewee’s experiences and assurances of confidentiality throughout the process. Factors concerning privacy and respect were also taken into consideration throughout the whole process.

### Data Analyses

The data collected were thoroughly analysed by reading and re-reading the transcripts in a bid to identify the key themes. Themes here refer to a patterned response or significant phrases that were frequently used by the interviewees (Edhlund and McDougall 2016). An inductive thematic analysis was used as the primary strategy because of its grounding in the data. The analysis helps to build a set of themes by looking for patterns and meanings produced in the data and then labelling and grouping them in connection with the theoretical framework of the research (Frost 2011). The following provides an overview of the key phases used to analyse the data.

#### Phase 1

The interview transcripts were carefully read by both authors, independently, in an effort to understand each narrative as a whole. The second author is a Muslim academic of South Asian heritage with expertise in issues of equality and diversity. Each interview transcript was analysed to identify the key themes and related examples. Notes and observations were exchanged after first round of independent analysis to identify if similar themes were attained. In a few instances of varied interpretations, the interview transcripts were re-read independently to revisit the analysis. This process was deemed useful to reduce personal or subjective bias. Moreover, considerable time was spent in reviewing the literature and comparing the emerging themes with previous findings, while also looking out for new themes to inform and add value to the existing literature.

#### Phase 2

After making a tentative list of themes, the interview transcripts were read carefully again to establish that nothing vital or relevant was missed. Redundancies were removed, and a refined list of themes was produced. The intersectionality framework acted as an aid to understand how gender, ethnicity, religion, and other dimensions of identity affected Muslim women’s career. Also, it helped to identify how different themes jelled together which helped to understand the different experiences these women faced.

#### Phase 3

Our analysis was further validated and cross-verified through the use of Nvivo 10.0, a qualitative software by QSR Australia used for computerised classifying, sorting, and arranging information. The purpose of using this software is to assist researchers in systematically finding themes and patterns in the qualitative data (Smith 2010). Coding was used to support analysis which assisted the researchers in thinking about the data, keeping track of decisions made, and building a case supported by the data and its frequency (Bazeley and Jackson 2013). Each theme was identified from the questions we asked the participants (e.g., gender, ethnicity, religion, and family status), then the focused codes were specifically identified around those themes (e.g., religion: negative comments, indirect discrimination).

We imported the transcripts into NVivo 10.0. Each coder (i.e., each co-author) started the focused coding phase by coding each transcript line-by-line. We also used NVivo to calculate interrater reliability and average Cohen’s Kappa was found to be .94. Additionally, we noted concepts and linkages to the literature review. Each researcher coded a transcript separately and then went through each transcript together and discussed whether each code represented intersectionality and if the codes actually captured what the participants were trying to convey. We identified six general themes and several focused codes within each theme, all of which reflected the overarching theme of intersectionality based on our conceptual analysis and the interview data. This overarching theme cuts across all the themes that emerged in the data to best explain how the overall process of intersectionality happened in this sample of South Asian heritage Muslim women.

## Results

The present study explores how women from South Asian Muslim heritage continue to face significant challenges related to their gender, ethnicity, and religion. Although discrimination is not blatant in most instances, it has an impact on these women’s employment and career. In the following, we report the key themes and some of prototypical responses that emerged from our interviews. Table 1 provides demographic profile of each participant, and these IDs are used to demarcate the source of each quote. Table 2 summarises the key themes and codes that reflect the participants’ experiences and perspectives in organisations. Although all themes were mentioned at least once by every participant, Table 2 further details the number of times across all interviewees that each theme was recounted.

**Table 1:** Participants’ characteristics

Participant ID	Age	Marital status	Organisational sector	Position
1	45–59	Married	Banking & Finance	Manager
2	20–29	Single	Telecommunications	Supervisor
3	30–44	Married	Engineering	Manager
4	20–29	Single	Science	Supervisor
5	30–44	Married	Government	Supervisor
6	30–44	Married	Charity	Manager
7	30–44	Married	Government	Manager
8	20–29	Single	Retail (Gifts)	Supervisor
9	30–44	Divorced	Charity	Leader
10	45–59	Married	Education	Leader
11	45–59	Married	Banking & Finance	Manager
12	30–44	Married	Charity	Leader
13	45–59	Married	Retail (Beauty)	Leader
14	45–59	Lesbian	Information Technology	Leader
15	45–59	Married	Education	Leader
16	30–44	Married	Marketing	Leader
17	30–44	Married	Education	Manager
18	30–44	Married	Charity	Leader
19	45–59	Divorced	Law	Manager
20	30–44	Married	Charity	Manager

Leader refers holding a chief executive role or an entrepreneurial position, Manager, to holding a middle-to-senior management position, Supervisor, to being in a responsible position but at the lower end of the organisational hierarchy

**Table 2:** Analysis of the interviews

Theme Focused code	Examples	Instances^a^
Gender Bigoted remarks; sexist treatment	“When I took the job working with the community and development as a chairperson, I received a lot of negativity from my male counterparts and senior colleagues. Six months into the job all I got was ‘women cannot do this job’ and I was never taken seriously.” (ID 10) “Sometimes I found the expectation for a woman was to make tea and coffee in the boardroom.” (ID 14)	120
Ethnicity and Religion Negative comments; indirect discrimination	“Senior colleagues would make inappropriate remarks, such as ‘oh it’s Ramadan, I hope you don’t pull any sickies.’” (ID 8) “In general, I think racism does exist within different organisations but it is difficult to prove and you need to be 100% sure.” (ID 13)	62
Perceptions; Cultural indicators	“Being an ethnic minority woman has helped me to progress in my career. To be honest it’s down to your appearance and how you speak. Because I am westernised in my appearance and how I come across is not much of a hindrance. Say if I was to wear a hijab or a shalwar qameez [traditional South Asian trousers and top], then I may be perceived differently.” (ID 6)	69
Family Marriage and children; family traditions	“I do think Muslim women are held back because of family traditions; this stops some of them to apply for promotions.” (ID 13) “In the early age, say 20s, when many women are going to university or starting their career, in some Muslim families, women are expected to get married and have children—straight after their studies.” (ID 12)	71
Intersectionality Overlapping of gender, ethnicity and religion	“What I found was all males recently had the opportunity to attend the conference but I was not allowed to go. I think the CEO was probably thinking how an Asian woman is going to present herself in a male-dominated environment.” (ID 15) “Gender doesn’t act alone but race also has a part in it if you are from an ethnic minority group. I was a race relations officer, I could not carry out certain tasks and I was working with two Black females and one White male. The male was allowed to carry out the tasks that were specified in the job description but the two ladies and I could not carry out what was stated in our job description.” (ID 10)	57
Agency Social support; confidence; training	“When I got married my career carried on, I have a very supportive spouse, as well as having two children. Alongside my job I am currently doing my PhD part-time “(ID 17). “The biggest challenge I faced as a Muslim woman was having to juggle with work life balance and the lack of confidence. I was always unsure if I could take the business to the next level.” (ID 15)	59

^a^All themes were expressed by all 20 interviewees at least one time. Instances reflects the number of discrete occurrences across all participants

### Gender

As expected, gender is a recurring theme in the organisational experiences, issues, and perspectives of Muslim female leaders, managers, and supervisors. Participants explained how gender was a key element affecting their treatment at work. For example, the following participant, who works in a highly male-dominated financial sector as a regional manager, explains how she was a victim of a bigoted remark from her male colleague and how she dealt with the issue. She also explains how her senior colleagues did not take the matter seriously and found it amusing. Her story suggests that gender discrimination is still not taken seriously in some organisations.

When I got my role as a regional manager, I was the only woman at the time and the rest were men. I can remember this one colleague, a bit of a show off, and it was my first meeting, and we were waiting for the meeting to start. He said to me “Love, make us a brew.” I looked at my right and left shoulder and said to him, “Are you speaking to me or your wife?” At the time, I could see my colleagues glaring at us; I could have said worse, but I had to act in a professional manner and not let my guard down. I just brushed it off my shoulders and thought that I am going to expect worse situations than this one. The organisation didn’t do anything about it. To be honest, most of them found it funny (ID 1).

Another participant who works in the Information Technology sector as a leader explains how she was discriminated against because of her gender while she tried to progress in her career. She has experienced similar bigotry as prior interviewee. This sector is highly dominated by men.

In meetings I found that being a woman at a senior position, you will hardly get any credibility from your male counterparts and senior colleagues. Sometimes I found that the expectation for a woman was to make tea and coffee in the boardroom. Sadly, gender stereotype still exists even now (ID 14).

Although on a policy level gender equality is taken seriously by the UK government and organisations (Razzu 2014; Stratigaki 2005), it appears as though negative stereotypes and perceptions around gender are still tolerated in some firms.

### Ethnicity and Religion

In addition to gender, ethnicity and religion are important themes in the organisational experiences of Muslim female leaders, managers, and supervisors. These issues arise not only from ethnic and cultural background but also religion. Although ethnicity and religion are separate concepts, these have close intersection in Muslin women’s lives in the United Kingdom. The following participant, who is a manager working in the engineering sector which is highly dominated by men, explains that not having role models from her own cultural background has had a major impact on her career. The only method she found comfortable to gain support was using social media networks to build professional relationships with other people in her own field, which she did in her own time.

I didn’t really receive much support from any mentors because the mentors in the organisation are White males. To be honest it is a lonely journey; what I found was that you made more enemies than friends as you progressed in your career. I missed not having any ethnic minority women as managers or leaders in the organisation I work for, as I would have preferred a role model that could have coached and mentored me. The only support I received was through networking via social media which I did in my own time (ID 3).

In contrast, the following participant, who works as a manager in the marketing sector, experienced positive effects of gender and ethnicity. She claims that her ethnicity affected her in a positive manner and actually helped her to progress in her chosen career. This participant further explains how she has been fortunate in terms for which organisation she chose to work because her employer and colleagues actively encourage diversity in the workplace. Ethnic networking played a key role in her employment. She explains that if it was not due to her ethnicity, she may not have been successful in gaining her job or promotion. Therefore, she feels her ethnicity has had a positive impact on her career.

I don’t think my ethnicity has affected me in a negative way; rather it has affected me in a positive way. For example, when I worked for [organisation], I was given the opportunity to work within the diversity department. At the time, they [senior colleagues] thought I would fit well working with that team as a senior member because I came from an ethnic minority group. So they had this perception that I could bring new ideas and expertise to the team, and coming from an ethnic minority group it was an advantage to them as they wanted to promote diversity within the organisation (ID, 16).

Our results suggest that whereas some Muslim women faced negative experiences in the workplace, a few of these women had positive experiences due to their ethnicity or faith.

### Family Issues

This section provides examples of how Muslim women and their careers are affected by family issues. The following participant, who works in the training and education sector as a leader, explains how her family, particularly her father, was less supportive of her career and instead she was pressured to get married. She further explains how this issue did not discourage her but in fact made her more determined to pursue her career.

I wanted to study a law degree after completing my A-levels, but my father was against the idea because he wanted me to get married rather than study away from home. In my 20s, I was pressured to get married, and 8 years after having my children, I set up a catering business because my father owned his own restaurants. This meant that he had the relevant contacts. Little did I know he was less supportive of my business idea because he thought two young women—my sister and I—trying to run a catering business which is highly dominated by men is not going to be achievable. We started the business anyways making Indian food, and I left the business in 2007 which was worth 6.5 million at that time (ID 15).

Our study shows that although some Muslim families may not be supportive of women entering the labour market. However, the women we interviewed were prepared to use their agency and strategies to tackle such issues.

In contrast, a few of these women stated that they had supportive families which made it easier for them to progress in their careers. As an example, the following participant, who works in academia in a leadership position, states that:

Being a Muslim woman, I was lucky because both of my parents were educated and teachers themselves. So I received all the support I needed from my parents, including my husband. Whilst I had young children, I committed myself to jobs that offered flexibility, and once my children started to get older, I began to progress in my career (ID 10).

Although the general perception is that Muslim families restrict women from entering the labour market, there are indeed examples of Muslim women whose families encouraged and supported their career.

### Intersectionality

In this section, we provide examples of how gender, religion, ethnicity, and family status together affect ethnic minority women at work. The following participant, who is a manager for a charity organisation, explains how she was discriminated against by a male senior colleague from her own religion because of her gender and ethnicity. She further explains that some Muslim men bring traditional stereotypes, with which they have been brought up, into the workplace and thus treat women as subordinates. This example indicates the complexity of perceptions and challenges Muslim women continue to face. The example also suggests that Muslim women face cultural stereotypes from Muslim or ethnic minority men at work.

I work for a charity organisation, and women, especially those from ethnic minority groups, are under-represented in this sector. What you will see is that men from ethnic minority groups who are in senior positions will try to hinder your career. For example, my manager who is a Muslim once said to me, “Oh you got three children so I think for you to progress to the next stage will be difficult.” Furthermore, I was once told by my manager that I could not travel to promote my campaign because I needed my husband with me (ID 6).

The above account suggests that Muslim women also face discrimination at the hands of ethnic minority men, which is evidence of how their intersectional identity affects their careers. The intersection of faith and gender is used to discriminate against them not only by ethnic majority or White colleagues but also by ethnic minority men from the same religious background.

Another participant who works in the government sector as a manager suggests that discrimination exists in those organisations that are expected to be supportive of diversity and equality. She further explains that she had previously applied for a senior position but a White female colleague with less experience and qualifications was selected.

Previously, I applied for a trainee manager’s post, and I remember this White woman who had less experience and qualifications than me but she got the job. That really upset me and I was afraid to ask my manager as well; it was only later on that I found that this particular manager wanted someone who didn’t have to worry about childcare, and because I was working part-time, he thought I would not commit 100% to my job. His perceptions about Muslim women were that once they have children, they leave employment sooner or later (ID 5).

Furthermore, the following response makes it explicit that intersectionality of various forms of identity is still a recurring issue. This participant completed her degree and became a manager in the service sector selling electrical goods. Having worked part-time while she was studying, she explains that she was never a victim of race discrimination until she was promoted. She also highlights the pressures with which she has to cope outside the workplace.

When I worked in the retail sector which sells electrical items, what I found was, when I became a manager, I faced discrimination from a few customers. There was an issue where one or two customers said to me, “Oh we don’t want to talk to you because you may be a terrorist.” What they meant was that if you’re a Muslim, then that means you’re a terrorist. I wear a headscarf, and sometimes I think for that reason some customers would rather seek help from someone else rather than me—preferably someone from the White community. Also the other issues I face is at home. Even though my parents are supportive of my career choices, I feel that my father would have preferred me to pursue higher education and become a doctor or something along those lines eventually, whereas my mother is happy for me to work and settle down (ID 4).

However, the following participant, who works in a government sector organisation as a leader, explains how her gender and ethnicity had a positive impact on her career. This was due to the reason that her organisation by its very nature took diversity seriously.

Personally, I have never faced race or gender discrimination. That is due to working for a race equality council which addresses racial discrimination. My job role is about leading an organisation that gives advice to clients who have faced discrimination based on the grounds of any protected characteristics under the Equality Act 2010, especially race. It would be both ironic and a big mistake if a senior colleague or member of staff was to discriminate against me as I know what my rights are and would know exactly how to exercise them (ID 12).

Our results suggest that although Muslim women are likely to face a myriad of challenges due to their intersecting identities, a few of them had positive experiences in the workplace.

### Agency

Our interviews suggest that Muslim women struggle to adopt basic traits from home which White women are likely to adopt from a young age, such as being confident, assertive, and out-spoken. However, some of these women have used their unique resources, such as engaging in ethnic and social networks and training courses, as well as individual hard work, to progress in their careers. For example, a leader working in the technology company explains how taking training courses helped her to progress in her career:

Whilst I was working in several organisations, I did attend training courses to enhance my career. I did my training whilst I was working in organisations. I also built up a social network which was done in my own time. I had to be prepared to put myself forward (ID 14).

For a Muslim woman, family support is of paramount importance. As the following participant explains, if she did not receive much support by her family, it would have been difficult for her to progress in her career. Her ideal support is her husband who allows her to progress in her career rather than expecting her to stay at home and look after the family. The following participant works in the education section as a manager:

Prior to my marriage I had a lot of support from my parents regards to my career. The only thing I was restricted from was travelling because in Muslim families they are over-protective of their daughters. When I got married, my career carried on. I have a very supportive spouse, as well as having two children. Alongside my job, I am currently doing my PhD part-time (ID 17).

In contrast, the following participant, who works for a local city council as a manager, explains that she did not integrate with other communities until she started university. She further explains that she adopted basic skills later in her career. However, she believes that if she had these traits (e.g., being confident, outspoken, and assertive) from a young age she may have progressed in her career sooner rather than later in her life. In addition, Interviewee 16, who works as a manager in the marketing sector, states that the reason she does not suffer from any form of discrimination at work was that she had gained basic skills at home which helped her to become a strong individual. Here is the response of Interviewee 7 who works for the local city council as a manager:

One of things they need is self-belief and confidence. Ethnic minority women, especially those that are from Muslim background, are reserved. Organisations have become competitive; that means they [Muslim women] need to stand out when they go for job interviews. Being reserved is not good in these situations. Again, a majority of ethnic minority women struggle to integrate with other communities for several reasons (ID 7).

Our findings show that while Muslim women continue to face issues and challenges in the workplace, some of them are able to use their individual agency and strategies to respond to such issues.

## Discussion

Using an intersectional lens, our study provides insight into the fact that Muslim women still face barriers and challenges within and outside work organisations while it also documents their agency and strategies they use to overcome such issues and challenges. A key challenge that Muslim women faced while trying to progress in their careers was the lack of access to network and mentoring support within and outside the workplace. Some thought that they had to work harder than their White counterparts to gain the same level of recognition, and others had to cope with derogatory comments made about their gender or ethnicity.

Our study has highlighted the permeating influences of intersectionality from societal context and individual identity to the domain of work, employment, and leadership. Consistent with intersectionality theory, the present study has revealed the ways in which oppressive institutions such as racism and sexism are interconnected and cannot be examined separately from one another (Phoenix 2006; Veenstra 2013). Racism is linked to sexism in the more direct sense that ethnic minority women are likely to experience racial and gender oppression simultaneously (Castles 2000). Overall, the themes highlighted in our study are interconnected and overlapping, which points towards the relational interplay of different levels of diversity (Syed and Özbilgin 2009).

Crenshaw (1989) explains that women have multi-layered facets in life that they deal with and that there are no one-size-fits-all types of feminism. This study reveals that not all ethnic minority women are going to face the same sort of racism and sexism because some may face more than others. For example, our study has illustrated that Muslim women who choose to wear the hijab or practice their religion at work face generally more challenges than those women who adopt a western or mainstream appearance. However, there are also a few examples of women who gained opportunities at work due to their ethnic or religious connection, as well as Muslim women who were penalized for their assimilation into western culture.

Our study has shown that Muslim women in the United Kingdom are exposed to complex and refined forms of sexism and racism, that is, ones which are subtle and hard-to-prove. This is also noted by Purdie-Vaughns and Eibach (2008) who argue that one way or another, ethnic minority women are likely to experience discrimination in the workplace. Moreover, taking intersectionality into consideration based on ethnic minority women in leadership, Porter and Sweetman (2005) state that these women are likely to experience far more challenges and discrimination than do White women in leadership positions. However, Sanchez-Hucles and Davis (2010) note that ethnic minority women are likely to feel more isolated without mentors or a network of support and are less able to garner the help that they might need when facing extraordinary challenges in leadership positions.

Recent studies reveal that women from the Pakistani and Bangladeshi regions have heard derogatory comments about their religious dress at work and that Black Caribbean women, because of their gender and ethnicity, believe they have to work harder than their White counterparts do to get to top positions (Kumra and Manfredi 2012). The present results suggest that Muslim women are likely to face additional pressures from their local community and family which is likely to affect their progression at work. Our study also has shown that even though Muslim women are going into higher education more than before, the expectation is to get married and have children straight after their studies. This suggests that some of these women are not even getting the opportunity to step into the labour workforce. Even for those Muslim women who had the support of their family were restricted from participating in particular activities. For example, a few of the Muslim women in our study reported that they were not allowed to travel to a different part of the country until they were married. However, such issues did not discourage these women from entering the labour market; in fact, they used a range of strategies and resources to progress in their careers.

### Limitations and Future Research Directions

In terms of limitations, our study focused on Muslim women from South Asian heritage working in UK organisations. Their experiences and narratives may not be similar to Muslim women of other ethnic or nationality backgrounds (e.g., African Muslim women). Also, given the fact that only a small proportion of South Asian Muslim women are represented at leadership, managerial, and supervisory positions, our interviews were conducted with women who were working in diverse sectors, some of which are highly dominated by men. To gain a well-rounded understanding of the issues and challenges Muslim women face, it may be useful to interview them from sectors which are not as highly dominated by men. Also, it may be suggested, and is worth investigating by future scholars, that the extent of these challenges may be different for non-practicing Muslim women who may be perceived differently from more devout Muslim women. Indeed, wearing a hijab or headscarf may alter the way a Muslim woman is perceived or treated in the workplace. In our study, it is interesting to note that one woman spoke about the advantages of her ethnic minority status in her job performance. Future scholars may focus on similar examples of positive work experiences from being a minority ethnic or religious group. Another fruitful area is to examine the extent and implications of internal pressure these women face in living one way at work and another way at home.

### Practice Implications

We used an inductive thematic analysis to consider the multi-layered nature of factors such as gender, ethnicity, religion, and family status that affect ethnic minority women’s career. The intersectional lens used in our study may help scholars, policymakers, and organisational leaders to think differently and analyse critically the interconnected issues of identity, equality, and power at work (Ng and Sears 2010). It may also help in understanding how ethnic minority cultures may differ from western culture and why it is important to understand and accommodate different values (Cook and Glass 2013).

Our study has illustrated the complex nature of issues and challenges South Asian heritage Muslim women face in pursuit of employment and leadership positions in the United Kingdom. Even though rules and regulations are put in place within organisations, ethnic minority Muslim women still face subtle and refined discrimination at work (Goddard 2013). This suggests that organisations need to place greater priority on goals and indictors when it comes to diversity.

Our findings illustrate that, in organisations which actively encourage diversity, these women are far more likely to progress in their careers. Indeed, our study also highlighted the unique strategies these women used to overcome any obstacles in the way of their employment and career. For example, some Muslim women stood their ground while they faced discrimination at work, and a few of these women took the organisations for which they worked to the industrial tribunal, based on the fact they had a strong case against them. Indeed, these findings may help other Muslim women and researchers who wish to understand the issues and challenges these women face while they progress in their careers and how deal with them. In order for organisations to take an active approach when it comes to diversity, strategic networks may be set up to mentor, monitor and advise ethnic minority women in their careers, preferably by female role models of their own ethnic background. Also providing diversity training schemes, particularly at a board level, may help to reduce negative attitudes toward Muslim and other ethnic minority women. Furthermore, the UK government could encourage organisations by offering incentives for enabling and promoting ethnic minorities, particularly women at the board level.

### Conclusion

We have shown that women from South Asian Muslim heritage continue to face multi-layered issues and challenges in pursuit of employment and leadership, which they try to negotiate through their unique skills and agency. Our qualitative study has demonstrated that Muslim women still face a myriad of challenges in the UK workplace, even though rules and regulations are put in place to curb discrimination (Goddard 2013). It suggests that legal compliance is not enough to tackle the complex issues and challenges of intersectionality. Indeed, there are also other factors within and outside the workplace (such as lack of mentors, positive action, and cultural- and family-related issues) which affect Muslim women’s careers. However, such issues have not discouraged these women from progressing in their career but rather they clearly demonstrated how they tackled such challenges.

Indeed, some of these women were able to draw positive energy from their ethnicity, religion, and gender to progress in their careers. However, given significant gaps not only in employment and leadership but also in unemployment, organisational policies on diversity require further fine-tuning to enable equal and inclusive opportunities to ethnic minority women and other disadvantaged individuals in the workplace. In addition to holding the organisation and government responsible, it is important to consider the roles of family, religion, and ethnicity as dimensions which Muslim families/Muslim men may need to reform in order to enable successful development of Muslim women in leadership positions in western countries.

## Electronic supplementary material

ESM 1 (DOCX 17 kb)
