# Supplementary material for: Intersectionality at Work: South Asian Muslim Women’s Experiences of Employment and Leadership in the United Kingdom
Source: Sex Roles. 2017 Mar 25;77(7):510–22. doi: 10.1007/s11199-017-0741-3 (PMC5596051; doi:10.1007/s11199-017-0741-3)
Supplement: Supplementary file 1 — (DOCX 17 kb) [file 11199_2017_741_MOESM1_ESM.docx]

Online Supplement for Tariq, M., & Syed, J. (2017). Intersectionality at work: South Asian Muslim women’s experiences of employment and leadership in the United Kingdom. *Sex Roles*. Memoona Tariq, University of Huddersfield, Email: [memoona.tariq@hud.ac.uk](mailto:memoona.tariq@hud.ac.uk)

**Interview Guide**

1. Demographic questions about ethnicity, gender, religion, nationality, first language, age, legal status, family status and academic and professional qualifications:
2. Please, can you explain your current organisation and role? Also provide time spent in your current job.
3. Please, take me back through the history in your career that brought you to this position?
4. What type of roles did you undertake throughout your career?
5. Did you enrol in higher education or an adult course to progress in your career?
6. What is your view and understanding of a leader in the context of work and organisations?
7. To what extent has your gender (i.e., being a woman) affected you in achieving senior, managerial or leadership roles within your organisation?
8. To what extent has your ethnic background affected you achieving senior, managerial or leadership roles?
9. Did you ever feel being treated differently at work because of your gender or ethnicity?
10. To what extent have you faced any challenges in your career being a Muslim woman?
11. How did you overcome such issues and challenges that affected your career?
12. To what extent did your organisation encourage you to progress in your career?
13. How did they encourage you?

1. Did they encourage ethnic and gender diversity in the organisation?
2. What can organisations do more to help ethnic minority women to progress in their chosen careers?
3. Do you ever feel less favoured than your male counterparts or senior colleagues? Any stereotypes that you face?
4. Do you ever feel less favoured than your white female counterparts? Explain.
5. What kind of support did you receive from your organisation to develop yourself as a manager or leader (e.g., through mentoring, coaching or networking)?
6. Do you feel things may be done differently within organisations where ethnic minority women feel at ease to progress to managerial and leadership positions?
7. Is there anything else you would like to add before we finish this interview?
